# Case report: MRI changes of the inner ear in an MD patient with suspected immune dysfunction

**DOI:** 10.3389/fneur.2023.1220162

**Published:** 2023-09-07

**Authors:** Yurun Chen, Pengfei Zhao, Xin Ma, Tongxiang Diao, Lisheng Yu

**Affiliations:** ^1^Department of Otolaryngology, Head and Neck Surgery, People’s Hospital, Peking University, Beijing, China; ^2^Beijing Friendship Hospital, Capital Medical University, Beijing, China

**Keywords:** Meniere’s disease, immune and inflammatory responses, endolymphatic hydrops, MRI, pathophysiology

## Abstract

**Objectives:**

The primary objective of this study was to present the progressive changes from labyrinthitis to endolymphatic hydrops (EH) demonstrated in the inner ear MRI of a patient with MD and suspected immune dysfunction.

**Patient:**

This 31-year-old male was diagnosed with MD and suspected autoimmune diseases.

**Interventions:**

Immunosuppressants and biological agents.

**Main outcomes measures:**

Inner ear MRI images.

**Results:**

Changes in the patient’s progress revealed that inner ear immune and inflammatory changes might induce EH, which may eventually turn into MD.

**Conclusion:**

This case is the first documented case of MRI revealing progressive changes from inflammatory response to endolymphatic hydrops in the inner ear. It shows the correlation between MD and inflammation visually. It is of great significance to reveal the pathogenesis of MD to further assist in the guidance of treatment decision making.

## 1. Introduction

Meniere’s disease was first proposed by Prosper Menière in 1861 and is characterized by recurring spontaneous vertigo, fluctuating hearing loss, tinnitus, and aural fullness. The prevalence of MD is approximately 34–190 per 100,000 (1). Not all patients with MD showed every typical symptom at the early stage of onset. Approximately 50% of patients with MD sustained vertigo and hearing loss, 19% suffered only vertigo, and 26% presented only hearing loss (2). In this case, the patient first developed vertigo before the onset of fluctuating hearing loss, tinnitus, and aural fullness.

Studies have shown that the occurrence of Meniere’s disease (MD) may be related to inflammation and immune dysfunction. In fact, as early as 1916, Wittmacck et al. proposed that some dizziness and imbalance diseases might be caused by infection based on temporal bone anatomy (3). Previous studies by our team have shown that MD patients are often associated with poorer mastoid pneumatization, which suggests that factors such as long-term drainage disorders and repeated inflammatory stimulation caused by anatomical variations may play an important role in the occurrence and development of MD (4).

Approximately one-third of patients with MD are accompanied with immune dysfunction (5). In 1986, Brooks et al. found that 54% of patients with MD had increased levels of immune complexes in their circulating blood. Several studies have described MD associated with autoimmune diseases such as rheumatoid arthritis, systemic lupus erythematosus, or psoriasis (6). Alleman et al. believed that this confirmed increase was caused by a local immune response in the endolymphatic sac, which originates from infection or allergy rather than from autoimmunity.

However, there remains a lack of direct evidence for the underlying pathophysiological mechanism of MD induced by immune and inflammatory responses. The changes of the inner ear demonstrated by MRI of the patient in this case intuitively demonstrated the progressive changes from labyrinthitis to endolymphatic hydrops (EH), which is of important clinical significance for not only validating the relationship between immune inflammatory response and MD, but also for providing a new direction to reveal the pathophysiology of MD and guiding its diagnosis and treatment.

## 2. Case report

A 31-year-old male presented to a physician with repeated episodes of rotational vertigo that lasted for a number of hours over the past 4 years (2017-09). He also reported nausea and vomiting with no tinnitus or hearing loss during the attack. Three years ago (2018-06), the patient started to experience left sided fluctuating tinnitus and sensorineural hearing loss (Table 1), accompanied by vertigo and aural fullness. He suffered attacks two or three times a week, lasting from 15 min to 2 h each. His past medical history was unremarkable, with no family history of dizziness or autoimmune disease.

**Table 1:** The patient underwent Pure Tone Threshold Audiometry (PTA) and inner ear MRI since onset.

Date	Audiograms	MRI	
2018-06	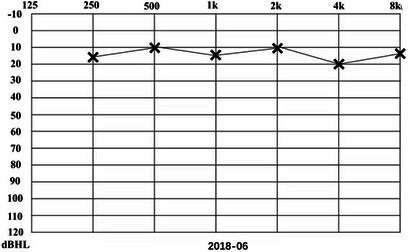		
2019-02	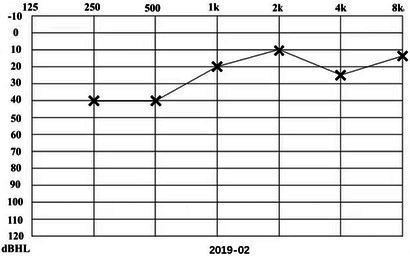	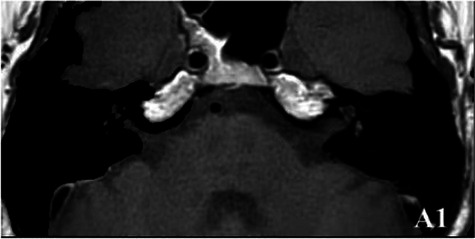	In March 2019, the left vestibular signal was higher than that in the contralateral in both the T1WI (A1) and 3DT2FLAIR sequences, whereas it was lower in the water imaging sequence (A2), indicating disruption of the blood-labyrinth barrier caused by inflammatory or hemorrhagic changes (A3).
2019-03		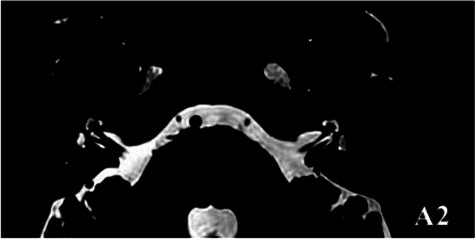	
		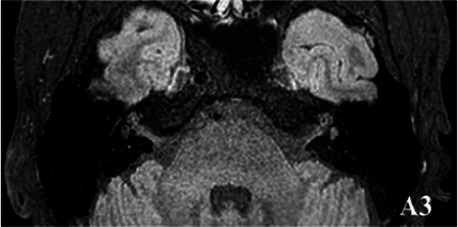	
2019-10	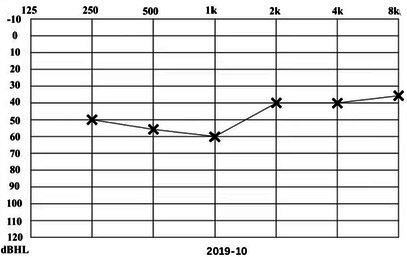	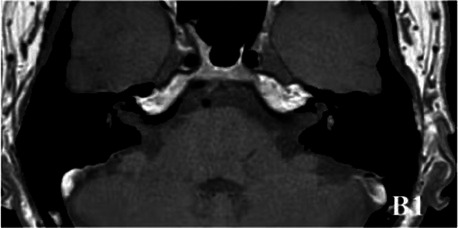	In October 2019, the signal of the left vestibular in the T1WI sequence remained higher than that in the contralateral (B1). In the 3D-T2-FLAIR sequence, the signals of the left vestibular and vestibular nerve were higher than those of the right (B2).
		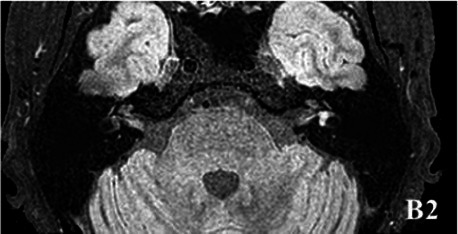	
2020-04		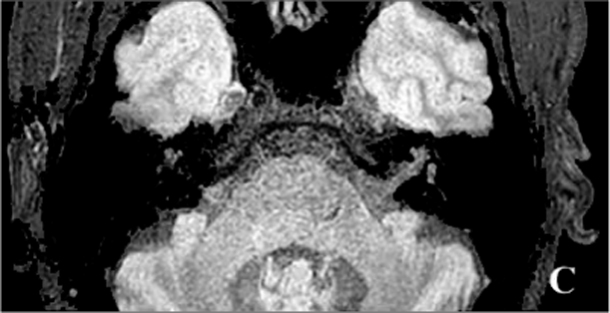	In April 2020, the signals of the left vestibular and vestibular aqueduct remained higher than that in the contralateral in the 3D-T2-FLAIR sequence. Nevertheless, the signal differences decreased (C), indicating that inflammatory lesions had subsided.
2020-12		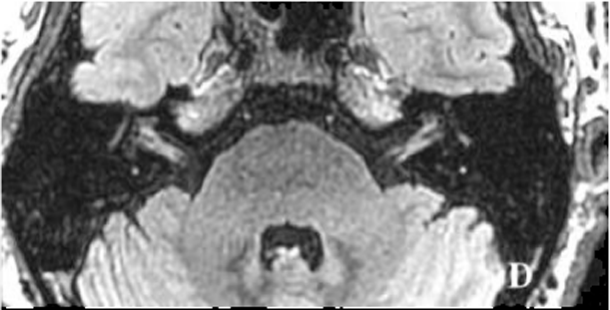	In December 2020, the left vestibular filling defect became larger under gadolinium angiography in the 3D-T2-FLAIR sequence, which was a sign of EH (D).
2021-03	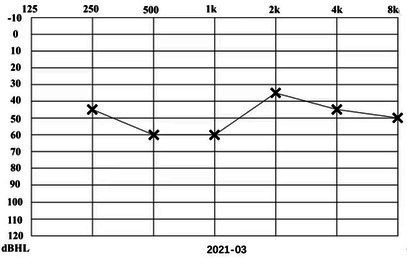		
2021-07	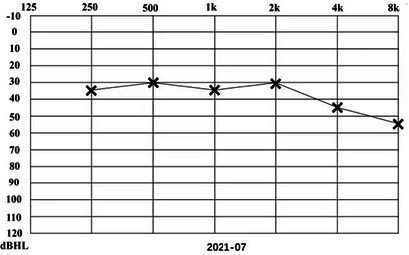	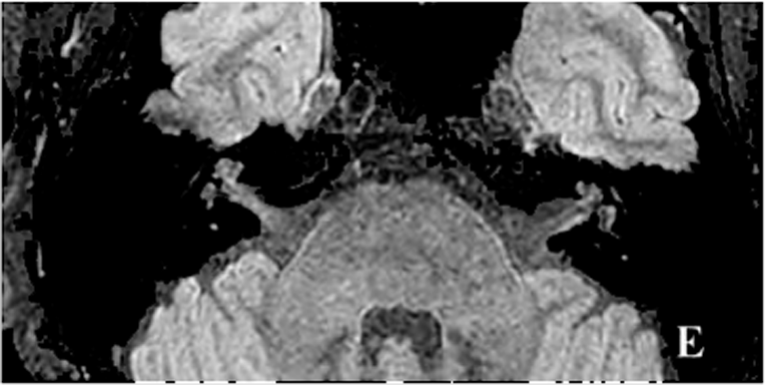	In July 2021, under gadolinium angiography in the 3D-T2-FLAIR sequence, left-sided EH improved (E).
2022-01	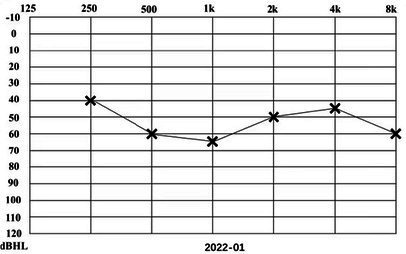		

There was no obvious abnormality in the video head impulse test (vHIT), while the caloric test showed left horizontal semicircular canal paresis (CP = 79%). This discrepancy indicates semicircular canal functions at normal head movement frequency and velocity ranges, but disfunctions at low frequency. No obvious abnormality was found in the electrocochleogram. No frequency of the left ear was elicited by distortion product otoacoustic emissions (DPOAE). Furthermore, ocular vestibular evoked myogenic potential (oVEMP) showed prolonged latency and low amplitude on the left-sided ear. Moreover, a lower amplitude on the left side was observed by cervical vestibular evoked myogenic potential (cVEMP).

The vHIT test is less sensitive for the detection of semicircular canal hypofunction than the caloric test (7). Considering all of the above, this patient was diagnosed with MD (1). Because the patient was less sensitive to vasodilators, flunarizine, and betahistine mesylate, but more sensitive to hormones, the immunologists believed that autoimmune diseases should be taken into consideration. After receiving immunosuppressants and biological agents, including tacrolimus, interleukin-2, cyclosporine, Rituxan, and Tocilizumab injections since March 2019, the patient reported that vertigo occurred less frequently. During the course of treatment, the immunologists attempted to reduce the dose of prednisone twice (2019-03, 2019-10). However, after the reduction, the patient experienced vertigo similar to before the therapy. Thereafter, methylprednisolone and prednisone were used alternately up until now. After receiving glucocorticoids and immunosuppressant therapy, the patient was relieved from vertigo and stopped losing hearing.

The patient underwent inner ear MRI every 6 months since onset; all examinations were performed using the same apparatus. In March 2019, the left vestibular signal was higher than that in the contralateral in both T1WI (A1) and 3DT2FLAIR sequences, whereas it was lower in the water imaging sequence (A2), indicating a disruption of the blood labyrinth barrier caused by inflammatory or hemorrhagic changes (A3). In October 2019, the signal of the left vestibular in the T1WI sequence remained higher than that in the contralateral (B1). In the 3D-T2-FLAIR sequence, the signals of the left vestibular and vestibular nerve were higher than those of the right (B2). The differences between both sides became more remarkable, which suggested progression of the disease. In April 2020, the signals of the left vestibular and vestibular aqueduct remained higher than that of the contralateral in the 3D-T2-FLAIR sequence. Nevertheless, the signal differences decreased (C), indicating that inflammatory lesions had subsided. In December 2020, the left vestibular filling defect became larger under gadolinium angiography in the 3D-T2-FLAIR sequence, which was a sign of EH (D). In July 2021, under gadolinium angiography in the 3D-T2-FLAIR sequence, left-sided EH improved (E).

The inner ear MRI of the patient showed the progressive change from exudative inflammatory changes to EH. This revealed that inner ear immune and inflammatory changes might induce EH, which can eventually turn into MD.

## 3. Discussion

EH is currently believed to be the main pathophysiology of MD; approximately one-third of patients with MD may be associated with immune dysfunction (5). Frejo (8) divided unilateral and bilateral MD patients into five clinical subtypes. One of these is MD combined with autoimmune diseases. As for this patient’s long course of disease, sensitivity to hormone and immunosuppressant therapy, and the rise in blood IgG4, immune diseases should be taken into consideration. However, there was no direct evidence on the pathological mechanism of how the immune inflammatory response induced MD. The inner ear MRI of this patient is the first documented case illustrating the progressive development from immune inflammatory changes to EH in the occurrence of MD. We speculate that local immune inflammatory responses in the inner ear can cause destruction of the blood-labyrinth barrier and labyrinthitis, can induce microcirculation disturbances, and can eventually lead to EH.

## 4. Conclusion

This case is the first documented case of MRI revealing the progressive changes from an inflammatory response to endolymphatic hydrops in the inner ear. It visually shows the correlation between MD and inflammation. It is of great significance to reveal the pathogenesis of MD, and this can further assist in the guidance of treatment decision making.

## Data availability statement

The original contributions presented in the study are included in the article/supplementary material, further inquiries can be directed to the corresponding authors.

## Ethics statement

Written informed consent was obtained from the individual(s) for the publication of any potentially identifiable images or data included in this article.

## Author contributions

YC: data curation, formal analysis, and writing-original draft. PZ: data curation. XM: validation, supervision, and funding acquisition. TD: conceptualization, methodology, writing-review and editing, and funding acquisition. LY: project administration and funding acquisition. All authors contributed to the article and approved the submitted version.

## Funding

This work was supported by Peking University People’s Hospital Scientific Research Development Funds (RDL2021–14), Peking University People’s Hospital Scientific Research Development Funds (RDY2021–25), and the National Key Technologies Research and Development Program of China (2020YFC2005200).

## Conflict of interest

The authors declare that the research was conducted in the absence of any commercial or financial relationships that could be construed as a potential conflict of interest.

## Publisher’s note

All claims expressed in this article are solely those of the authors and do not necessarily represent those of their affiliated organizations, or those of the publisher, the editors and the reviewers. Any product that may be evaluated in this article, or claim that may be made by its manufacturer, is not guaranteed or endorsed by the publisher.

## Abbreviations

MD: Meniere’s disease
EH: Endolymphatic hydrops
